# Toxicological evaluation of the aqueous stem bark extract of *Bridelia ferruginea* (Euphorbiaceae) in rodents

**DOI:** 10.1515/intox-2015-0014

**Published:** 2015-06

**Authors:** Olufunsho Awodele, Kennedy Iliya Amagon, John Agbo, Majeti Narasimha Vara Prasad

**Affiliations:** 1 Department of Pharmacology, Therapeutics & Toxicology, College of Medicine, University of Lagos, Lagos, Nigeria; 2 Department of Pharmacology, Faculty of Pharmaceutical Sciences, University of Jos, Nigeria; 3 Department of Plant Sciences, Gachhibowli, University of Hyderabad, Hyderabad Telangana, India

**Keywords:** *Bridelia ferruginea*, hematology, biochemistry, oxidative stress, sperm count

## Abstract

*Bridelia ferruginea* is a woody shrub that grows in the Savannah or rain forests of Africa and has traditionally been used to treat diabetes, arthritis and boils. Despite all these uses, extensive toxicological evaluation has not been carried out. The aim of the present investigation was to evaluate the sub-chronic toxicological effects of the stem bark aqueous extract of *Bridelia ferruginea* in rats. The lethal dose (LD_50_) was determined using probit analysis and graded doses of the extract (250–4000 mg/kg) were administered to the animals via oral and intraperitoneal routes and observed for mortality, behavioral changes and signs of toxicity. Sub-chronic toxicity study was carried out at doses of 1 000, 2 000 and 4 000 mg/kg administered daily for 60 days. The animals were sacrificed after 60 days. Blood was collected for biochemical (renal and hepatic), hematological, oxidative stress, sperm and histopathological examinations, using standard methods. LD_50_ of the extract was estimated as >4 000 mg/kg orally; neither significant visible signs of toxicity nor mortality were observed. There were no significant differences in the animals and organ weights, hematological and biochemical parameters in the treated groups compared to the control group. However, a significant increase (*p*<0.05) in the level of lipid peroxidation and a significant (*p*<0.05) decrease in sperm count were observed in the treated animals compared with the control group. The stem-bark aqueous extract of *Bridelia ferruginea* was found to be relatively safe, though it has the potential to cause lipid peroxidation and damage sperm quality and should thus be used with caution.

## Introduction

Medicinal plants play an important role in health care in Africa. The use of these medicinal plants, however, is not devoid of danger to health and exposes users to the risk of toxicity as well as unwanted side effects (Sonhi, 2002; Hilaly *et al*., 2004; Maïga *et al*., 2005). *Bridelia ferruginea* Benth (Euphorbiaceae) is a woody shrub that grows in the Savannah or rain forests of Africa (Njamen *et al*., 2012) and is traditionally used to treat diabetes (Onunkwo *et al*., 1996), arthritis (Olajide *et al*., 2007) and boils (Talla *et al*., 2002). The aqueous extract of the stem-bark of this plant contains quinones, gallic and catechic tannins, alkaloids, sterols, polyterpenes, polyphenols, reducing compounds, flavonoids and saponosides (Nene-Bi *et al*., 2009).

The recent study of Edeoga *et al*. (2005) has shown that the active constituents in medicinal plants are chemicals and the therapeutic uses of medicinal plants should be done with consciousness of using phytochemicals. A high percentage of people in both developed and developing countries use medicinal plants for therapeutic purposes (Awodele *et al*., 2011) and this is consistent with the estimation of the World Health Organization (WHO) that about 78–80% of the world’s populations rely on medicinal plants for their primary health care (Farnsworth *et al*., 1985). One of the major reasons that may be responsible for the increased use of medicinal plants is the notion that all herbal products are safe and effective (Farnsworth and Soejarto, 1985; Soejarto, 1989). However, consumption of herbal products or traditional medicines by various ethnic groups involves challenges and drawbacks, including several adverse effects, sometimes life-threatening, thus putting into question the safety of herbal remedies (Soejarto, 1989; Elvin-Lewis, 2001). Instances of adulteration, inappropriate formulations or lack of understanding of drugs and plant interactions or their uses leading to adverse reactions, sometimes life-threatening or lethal to patients, have been reported (Ernst, 1998). Thus, contrary to the popular belief that herbal medicines are safe, the use of herbal remedies can pose serious health risks (Wood, 2002).

Although a substantial number of scientific research papers have revealed the therapeutic activities of many African plants, there is paucity of data on the toxicity of these plant materials (Abalaka *et al*., 2009). In light of the report by Wood (2002), determination of both the efficacy and safety of medicinal plants are germane to their quality assurance. Hence, the guideline for toxicological evaluation of medicinal plants, as stipulated by the WHO, entails holistic toxicological assays so as to be able to make a reasonable conclusion on toxicity profiles.

*Bridelia ferruginea* (Euphorbiaceae) is a shrub or straggly tree of about 15m high with crooked bole and an up to 1.8m girth. It is commonly found in the savannah and its common names in Nigeria include: Kirni, Kimi (Hausa), Maren (Fulani), Iralodan (Yoruba), and Oha, Ede, Ola in Igbo. Ethnobotanical literature and folklore uses describe ulcer-protective and anti-diarrheal effects of the aqueous stem bark (Akuodor *et al*., 2012); prevention of metabolic syndrome in type 2 diabetes (Bakoma *et al*., 2013); purgative and vermifuge activities (Cimanga *et al*., 1999); milk coagulation and formulation of traditional gargle “ Ogun efu” (Orafidiya *et al*., 1990); antimicrobial activities against some micro-organisms known to cause enteric and secondary upper respiratory tract infections (Magistretti *et al*., 1988); anti-inflammatory activity (Olajide *et al*., 1999) and potential for water treatment (Kolawole and Olayemi, 2003).

Despite all these therapeutic uses of *Bridelia ferruginea,* extensive toxicological evaluation as stipulated by the WHO has not been carried out. However, Bakoma *et al*. (2013) performed a 28-day sub-acute study which produced interesting results, though devoid of extensive toxicological parameters. In view of this, the study was aimed at extensively evaluating the toxicity profile of *Bridelia ferruginea* using animal models.

## Methods

### Plant collection/extract preparation

Fresh stem bark of *Bridelia ferruginea* was collected from Odofin Agbebi farm, close to Ilare township secondary forest, in the Ikire Local Government Area of Osun State, Nigeria. Botanical identification and authentication were performed in the Department of Botany, Faculty of Science, University of Lagos, Lagos, Nigeria. A voucher specimen (LUH 5761) was deposited in the herbarium of the University of Lagos, Akoka, Yaba, Lagos.

Fresh stem bark of *Bridelia ferruginea* was air dried for about 9 days and the dried materials were ground to fine powder using a specialized grinding machine at the Department of Pharmacognosy, College of Medicine, University of Lagos, Lagos, and soaked in boiled distilled water overnight (100 g in 2 L). The extract was decanted 24 h later and the filtrate evaporated to dryness in an oven for 5 days at 45 °C, giving it a dark brown color. The calculated yield value was determined to be 25%. The dried extract was weighed and reconstituted in distilled water just before administration to experimental animals, to obtain a stock solution of 100 mg/mL.

### Animals

Male Wistar albino mice (average weight 20 g) and male albino rats (average weight 130 g) used in this study were obtained from the Laboratory Animal Center of the College of Medicine, University of Lagos, Lagos, Nigeria. The animals were maintained under standard environmental conditions (24–25 °C, 12h/12h light/dark cycle) and were fed on Pfizer standard rodent pellet diet and water *ad libitum.* The investigation conformed to the Guide for the Care and Use of Laboratory Animals published by the U.S. National Institutes of Health (2008) for studies involving experimental animals. The use of mice in acute toxicity study and rats in chronic toxicity study is a standard toxicological procedure. The purpose of the acute toxicity was to investigate the acute lethality of the agent and mice are often used because of their nature and strength compared to rats (Awodele *et al*., 2010). Two different routes of administration (oral and intraperitoneal) were used for the acute toxicity study as it is conventional and purposeful to evaluate the effect of different routes on safety and development of toxicity following drug administration.

### Acute toxicity study

#### Oral acute toxicity

The mice were randomly divided into five groups of five animals per group. Graded doses of the extract (250, 500, 1 000, 2 000 and 4 000 mg/kg) were administered to the animals orally. The control group was administered 0.1 mL of distilled water orally. The mice were observed for 24 h post-treatment for mortality, behavioral changes (restlessness, dullness, agitation) and signs of toxicity.

#### Intraperitoneal acute toxicity

The mice were randomly divided into five groups of five animals per group. Graded doses of the extract 250, 500, 1 000, 2 000, and 4 000 mg/kg were administered intraperitoneally. The control group was administered 0.1 mL distilled water. The mice were observed for 24 h post-treatment for mortality, behavioral changes and signs of toxicity.

### Sub-chronic toxicity study

The rats were randomly allotted to four groups of 10 animals each. The animals received an aqueous extract of *Bridelia ferruginea* stem bark orally at doses of 1 000, 2 000 and 4 000 mg/kg daily for 60 days. The control group was administered 0.2 mL of distilled water via the oral route. The rats were weighed weekly throughout the duration of the experiment. The animals were closely observed for behavioral changes such as restlessness, hyperactivity and dullness, as well as for general morphological changes. Blood samples were collected at the end of the study for determination of serum oxidative stress parameters, liver enzymes biomarkers, urea, creatinine and hematological parameters. The epididymis was processed for sperm analysis assays. Histopathological investigations of the kidney, liver, brain and heart were also carried out.

### Blood collection and organ samples

The animals were sacrificed on the 61^st^ day of the experiment. Each animal was placed in a closed receptacle containing cotton wool soaked with diethylether and placed in cages in a quiet area to minimize excitement and trauma until euthanasia was complete. To confirm death, each animal was monitored for the following signs: no rising and falling of the chest and no response to toe pinch. Blood samples were collected via ocular puncture with the aid of a capillary tube into EDTA (ethylenediaminetetraacetic acid) bottles and heparinized bottles for respective hematological and blood chemistry analysis. The kidney (left and right), liver, brain and heart were carefully isolated, weighed for histopathological examination and the epididymis was immediately removed for sperm quality analysis.

### Hematological analysis

Red blood cell (RBC) count, hemoglobin (Hb), mean cell volume (MCV), mean corpuscular hemoglobin (MCH), mean corpuscular hemoglobin concentration (MCHC), white blood cell (WBC) count, platelet (PLT), neutrophil (NET), pack cell volume (PCV), and lymphocytes (LYMP) were determined using standard methods and a fully automated hematology analyzer (pentra-XL 80, Horiba ABX, USA).

### Determination of liver enzyme biomarkers, urea and creatinine

The parameters of liver enzymes {aspartate aminotransferase (AST), alanine aminotransferase (ALT), alkaline phosphatase (ALP)}, urea and creatinine were determined using a fully automated clinical chemistry analyzer (Hitachi 912, Boehringer Mannheim, Germany).

### Measurement of the activity of serum antioxidant enzymes and malondialdehyde (MDA)

#### Determination of superoxide dismutase activity (EC 1.15.1.1)

The reaction mixture (3 mL) contained 2.95 mL 0.05 M sodium carbonate buffer pH 10.2, 0.02 mL of serum and 0.03 mL of epinephrine in 0.005 N hydrochloric acid. Serum superoxide dismutase activity was determined by its ability to inhibit the auto-oxidation of epinephrine determined by the increase in absorbance at 480 nm as described by Sun and Zigman, (1978). Enzyme activity was calculated by measuring the change in absorbance at 480 nm for 5 min. ∑= 4020 M^–1^ cm^–1^.

#### Determination of catalase activity (EC 1.11. 16)

Serum catalase activity was determined according to the method of Beers and Sizer as described by Usoh *et al*. (2005) by measuring the decrease in absorbance at 240 nm due to the decomposition of hydrogen peroxide in a UV recording spectrophotometer. The reaction mixture (3 mL) contained 0.1 mL of serum in phosphate buffer (50 mM, pH 7.0) and 2.9 mL of 30 mM hydrogen peroxide in phosphate buffer pH 7.0. An extinction coefficient at 240 nm hydrogen peroxide of 40.0 M^–1^ cm^–1^ was used for the calculation. The specific activity of catalase was expressed as moles of hydrogen peroxide reduced per minute per mg protein.

#### Reduced glutathione determination (EC 2.5.1.18)

The reduced glutathione content of serum as non-protein sulfhydryl, as described by Kadam and Abhang, (2013), was determined by adding 10% tricarboxilic acid to the sample and 1.0 mL of the mixture was treated with 0.5 mL of Ellmans reagent (19.8 mg of 5,5-dithiobisnitro benzoic acid in 100 mL of 0.1% sodium nitrate) and 3.0 mL of phosphate buffer (0.2 M, pH 8.0). The absorbance was read at 412 nm. Σ = 1.34 × 104 M^–1^ cm^–1^

#### Determination of glutathione-S-transferase

The cytosolic and mitochondrial GST activities were measured using 1 chloro-2,4-dinitrobenzene (CDNB) or 4-HNE as substrate as described by Kadam and Abhang (2013).

#### Determination of ascorbic acid (Vitamin C)

Concentration was measured according to the method described by Benderitter *et al*., 1998. Briefly, to 0.5mL of plasma 1.5 mL of 6% TCA was added and centrifuged (3500 g, 20 min). To 0.5 mL of supernatant 0.5 mL of DNPH reagent (2% DNPH and 4% thiourea in 9 N sulphuric acid) was added and incubated for 3 hours at room temperature. After incubation 2.5 mL of 85% sulphuric acid was added and color developed was read at 530 nm after 30 min.

#### Determination of lipid peroxidation (EC 202-974-4)

Malondialdehyde, an index of lipid peroxidation, was determined by adding 1.0 mL of the sample to 2 mL of (1:1:1 ratio) thiobarbituric acid 0.37%, 0.24 N hydrochloric acid and 15% tricarboxylic acid reagents. Tricarboxylic acid-thiobarbituric acid-hydrochloric acid reagents boiled at 100 °C for 15 min and were allowed to cool. Flocculent materials were removed by centrifugation at 3 000 rpm for 10 min. Absorbance was read at 532 nm against a blank. Malondialdehyde was calculated using the molar extinction coefficient for malondialdehyde-thiobarbituric acid complex of 1.56 × 105 M^–1^ cm^–1^.

The protein content was measured by biuret method as described by Gornall *et al*. (1949) with bovine serum albumin (BSA) as standard.

### Sperm analysis

Sperm analysis to assess seminal fluid for motility, count and morphology was carried out according to the method of Morakinyo *et al*. (2008). Briefly, laparotomy was done to expose the reproductive tract. The caudal epididymis was carefully isolated and minced with scissors to release the sperm. Each chamber of the hemocytometer was loaded with 10 microliters of the diluted sperm (1:20 dilution) and allowed to stand or settle for 5 minutes.

#### Sperm count

A 1 in 20 dilution of semen was carried out using sodium bicarbonate-formalin diluting fluid. An improved Neubauer ruled chamber was filled with the well-mixed diluted semen using a Pasteur pipette. The number of spermatozoa in an area of 2 sq mm was counted using the 10× objective of the microscope after 3–5 min. Estimation of the number of spermatozoa in 1 mL of fluid was done by multiplying the number counted by 100 000.

#### Sperm morphology

Sperm morphology was determined using eosin and nigrosin stain. Ten microliters of eosin and nigrosin were mixed with about 40 microliters of sperm suspension. The sperm suspension was incubated at 40 °C for about 5 minutes and re-suspended with a micropipette. Normal and abnormal spermatozoa were examined using the 40× objective of the microscope. Morphological abnormalities were classified as headless sperm, banana head, bent neck and bent tail. Estimation of the percentage of normal and abnormal morphology was done from the counting of hundred spermatozoa.

#### Sperm motility

Sperm motility was assessed by placing 10 μl of sperm suspension on a slide for microscopic evaluation at a magnification of 400×. About 200 sperm cells were examined and classified as either motile or immotile. Assessment of motile sperms was calculated as mean motile sperm number ×100/total number of sperm.

### Histopathological assay

The various tissues obtained from the experimental animals fixed in 10% formol-saline were dehydrated in graded alcohol, embedded in paraffin, and cut into 4–5 μm thick sections. Hematoxylin-eosin was used to stain the sections for photomicroscopic assessment using a Model N-400ME photomicroscope (CEL-TECH Diagnostics, Hamburg, Germany) (Gornall *et al*., 1949; Morakinyo *et al*., 2008). Slides were examined using ×40, ×100, and ×400 objectives. The liver, kidney, heart, and the brain of the animals were weighed and fixed in 10 % formalin in properly labeled bottles, processed routinely and embedded in paraffin wax. Sections of 5 μm thickness were cut, stained with hematoxylin and eosin and examined using a Model N-400ME photomicroscope (CEL-TECH Diagnostics, Hamburg, Germany) by a pathologist.

### Statistical analysis

Results were expressed as mean ± SEM. The data were subjected to one-way analysis of variance (ANOVA) test and the differences between samples were determined by Dunnett's multiple comparison tests, using the graph pad prism statistical software (GraphPad Software Inc., CA, USA). Results were considered significant at *p*<0.05.

## Results

The aqueous stem bark extract of *Bridelia ferruginea* did not produce any mortality when administered orally at various doses of 250 mg/kg to 4 000 mg/kg, but 2 hours post treatment reduced locomotion and dullness were observed in some animals treated with higher doses of 2 000 and 4 000 mg/kg (Table 1).

**Table 1:** Acute (oral) toxicity study in mice after 24 hrs of administration of aqueous stem bark extract of *Bridelia ferruginea.*

Group	Dose (mg/kg)	D/ T *	Period of signs observation (hr)	Signs of toxicity observed
A	0.2 mL (H_2_O)	0/5	24	No toxic changes observed.
B	250	0/5	24	No toxic changes observed.
C	500	0/5	24	No toxic changes observed.
D	1000	0/5	24	No toxic changes observed
E	2000	0/5	24	Slight dullness was observed in some animals in the first 2 hrs
F	4000	0/5	24	Slight dullness was observed in some animals in the first 2 hrs

* D/ T: Number of mice deaths/total number of mice (n=5).

The aqueous stem bark extract of *Bridelia ferruginea* did not produce any mortality when administered intraperitoneally *(i.p)* at various doses of 250 mg/kg to 4 000 mg/kg, but 2 hours post treatment reduced locomotion and dullness were observed in some animals treated with higher doses of 2 000 mg/kg (Table 2).

**Table 2:** Acute (*i.p.*) toxicity study in mice after 24 hrs of administration of aqueous stem bark extract of *Bridelia ferruginea*.

Group	Dose (mg/kg)	D/ T *	Period of signs observation (hr)	Signs of toxicity observed
A	0.25 mls (H_2_O)	0/5	24	No toxic changes observed.
B	250	0/5	24	No toxic changes observed.
C	500	0/5	24	No toxic changes observed.
D	1000	0/5	24	No toxic changes observed.
E	2000	0/5	24	Slight dullness was observed in the first 2 hrs

* D/ T: number of mice deaths/total number of mice (n=5).

There were no significant differences (*p*≥0.05) in the weight of the animals (day 0 to day 60) when compared with controls (Table 3).

**Table 3:** Effects of aqueous stem bark extract of Bridelia ferruginea on the body weights of rats.

Parameter (g)	Control	1 000 mg/kg	2 000 mg/kg	4 000 mg/kg
Weight at day 0	108.2±4.630	134±1.703	139.6±2.581	138.6±4.686
Weight at day 60	137.8±12.142	178.6±9.760	150.6±5.464	151±8.118
Weight gain	29.6	44.6	11.0	12.4

n=5; Data represented as mean ± SEM

There were slight variations but no statistically significant differences (*p*≥0.05) in the weight of the organs (heart, liver, kidneys) per 100 g body weight when compared with controls (Table 4).

**Table 4:** Effects of aqueous stem bark extract of Bridelia ferruginea on weight of organs of rats per 100g body weight.

Parameter (g)	Control	1 000 mg/kg	2 000 mg/kg	4 000 mg/kg
Heart	0.354±0.032	0.36±0.281	0.398±0.020	0.452±0.013
Liver	4.06±0.1600	4.24±0.237	4.50±0.1870	4.78±0.205
L.Kidney	0.318±0.028	0.338±0.012	0.402±0.040	0.452±0.066
R. Kidney	0.346±0.031	0.348±0.009	0.384±0.024	0.442±0.043

n=5; Data is represented as mean ± SEM

Table 5 shows a non-significant (*p*≥0.05) increase in RBC, HGB, PLT, MCH, and PCV, except at 4 000 mg/kg where a decrease was observed compared to the control. The results also showed a non-significant (*p*≥0.05) decrease in MCV and MCHC (except at 4 000 mg/kg where an increase was observed), compared to the control. The lymphocyte percentage (%) shows a significant (*p*<0.01) increase at 4 000 mg/kg compared to the control, while a significant (*p*<0.05) increase in WBC was observed in the group of animals administered 2 000 mg/kg, compared to the control. The neutrophil percentage (%) showed a significant (*p*<0.05) increase at 4 000 mg/kg compared to the control. The PCV showed a non-significant increase compared with the control except for the 4 000 mg/kg dose where the value was lower than in the control.

**Table 5:** Effects of aqueous stem bark extract of Bridelia ferruginea on the hematological parameters in rats.

Parameter (g)	Control	1 000 mg/kg	2 000 mg/kg	4 000 mg/kg
RBC (×10^12^)	7.766±0.142	8.326±0.186	7.846±0.130	8.022±0.194
HGB (g/dl)	14.020±0.245	14.12±0.226	14.04±0.215	14.06±0.282
MCV (fl)	17.840±0.156	17.080±0.156	17.56±0.301	17.62±0.102
MCH (pg)	61.900±0.578	63.180±3040	62.20±0.275	61.82±0.455
MCHC (g/dl)	28.200±0.208	27.420±4030	27.66±0.355	28.56±0.163
PCV (×10^9^)	49.560±0.697	50.340±0.398	49.70±0.584	49.18±1.610
PLT (×10^9^/L)	623.00±11.88	668.40±14.39	658.00±17.7	673.00±8.21
WBC (×10^9^)	9.820±0.1772	8.300±0.2074	12.42 ±0.368*	9.240±1.031
NEU (%)	32.480±0.270	34.240±0.287	35.04±1.102	36.66±1.109*
LYMP (%)	52.700±1.773	58.360±0.445	57.38±2.398	63.90±1.549**

Results are presented as Mean ± SEM (n=5)

* *p*-value <0.05 as compared to the control

** *p*-value <0.01 as compared to the control. RBC (Red Blood Cells), HGB (Hemoglobin), MCV (Mean Corpuscular Volume), MCH (Mean Cell Hemoglobin), MCHC (Mean Cell Hemoglobin Concentration), PCV (Pack Cell Volume), PLT (Platelet), WBC (White Blood Cells), NEU (Neutrophils), LYMP (Lymphocytes), SEM (standard error of mean).

The data in Table 6 show the effects of aqueous stem bark extract of *Bridelia ferruginea* on oxidative stress parameters. The results showed a statistically significant reduction (*p*<0.001) in superoxide dismutase (SOD) levels in all the treated groups when compared to the control, with group 3 (4 000 mg/kg) exhibiting the lowest mean. Catalase (CAT) showed no statistically significant (*p*≥0.05) difference in any of the treated groups compared with the control, though levels decreased with increasing dose.

**Table 6:** Effects of aqueous stem bark extract of *Bridelia ferruginea* on oxidative stress parameters in rats.

Parameter (g)	Control	1000 mg/kg	2 000 mg/kg	4 000 mg/kg
SOD (μ/mg)	114.7±0.198	47.55±0.253***	79.134±0.88***	43.25±0.267***
CAT (μ/mg)	750.1±7.223	687.2±8.875	611.3±11.46	561.7±35.38
GSH (μ/mg)	11.88±0.360	8.924±0.545**	5.968±0.427***	5.064±0.533***
GST (μ/mg)	34.54±0.059	14.320±.076**	23.83±0.267	13.02 ±0.081**
VITC (μ/mg)	82.90±5.056	49.47±2.469***	51.43±2.58***	35.57±0.986***
MDA (μ/mg)	1.260±0.231	2.802±0.139***	4.952±0.13***	6.764±0.202***

Results are presented as Mean ± SEM (n=5)

* *p*<0.05 as compared to the control

** *p*<0.01 as compared to the control

*** *p*<0.001 as compared to the control. SOD (superoxide Dismutase), CAT (Catalase), GSH (Glutathione), GST (Glutathione Transferase), VitC (Vitamin C), MDA (Malondialdeheyde), SEM (standard error of mean).

Glutathione (GSH) showed a significant decrease (*p*<0.01) in group 1 (1 000 mg/kg) compared to the control and a further decrease (*p*<0.001) in groups 2 and 3 compared to the control. Glutathione transferase (GST) levels showed a significant reduction (*p*<0.01) in groups 1 and 3 when compared to the control, while Vitamin C (Vit C) levels showed a statistically significant reduction (*p*<0.001) in all the treated groups when compared to the control, with the least reduction in group 3 (4 000 mg/kg). The MDA results showed a dose dependent significant increase (*p*<0.001) in all the treated groups compared with the control.

Table 7 shows the effects of aqueous stem bark extract of *Bridelia ferruginea* on liver enzymes and renal functions. Asparatate transaminase (AST) levels show a non-significant (*p*≥0.05) decrease in all the treated groups when compared to the control. However, alanine transferase (ALT) levels show a statistically significant (*p*<0.001) decrease in all the treated groups when compared to the control. Alkaline phosphatase (ALP), and creatinine (CRE) levels showed no statistically significant (*p*≥0.05) difference in any of the treated groups when compared to the control. Urea (URE) levels showed a significant (*p*<0.001) decrease in all the treated groups when compared to the control.

**Table 7:** The effects of aqueous stem bark extract of Bridelia ferruginea on biochemical parameters in rats.

Parameter (g)	Control	1000 mg/kg	2 000 mg/kg	4 000 mg/kg
AST (μ/L)	34.22±0.860	32.22±0.860	33.24±0.092	33.98±0.259
ALT (μ/L)	25.14±0.050	24.22±0.860***	23.24±0.092***	23.62±0.086***
ALP (g/L)	29.32±0.058	29.90±0.158	30.20±0.237	30.70±0.074
UREA (mmol/L)	8.58±0.3141	6.64±0.1699***	6.46±0.1208***	6.84±0.1503***
CRE (μmol/L)	43.45±0.057	43.20±0.070	43.72±0.132	43.25±0.036

Results are presented as Mean ± SEM (n=5)

* *p*<0.05 as compared to the control

** *p*<0.01 as compared to the control

*** *p*<0.001 as compared to the control. AST (Asparate Transaminase), ALT (Alanine Transaminase), ALP (Alkaline Phosphatase) CRE (Creatinine), SEM (standard error of mean).

Table 8 shows the effects of aqueous stem bark extract of *Bridelia ferruginea* on sperm quality of rats. The sperm motility shows a statistically not significant (*p*≥0.05) decrease in all the treated groups when compared to the control. Sperm morphology and abnormality also exhibited non-significant (*p*≥0.05) increases in all the treated groups compared to the control. The sperm count results showed a significant reduction (*p*<0.05) in all the treated groups except the 4 000 mg/kg group, compared to the control.

**Table 8:** Effects of aqueous stem bark extract of *Bridelia ferruginea* on sperm quality of rats.

Parameter (g)	Control	1 000 mg/kg	2 000 mg/kg	4000 mg/kg
Motility (%)	70±4.785	55±6.325	59±4.00	67±3.391
Morphology (%)	7.2±1.715	8.6±1.400	9.8±1.158	8.4±0.927
Sperm count (×10^6^)	56.25±2.305	40.25±1.447*	44.75±3.77*	56.25±3.0870

Results are presented as Mean ± SEM (n=5)

* *p*<0.05 as compared to the control

** *p*<0.01 as compared to the control

*** *p*<0.001 as compared to the control.

### Histopathological assay

**Figure 1:**
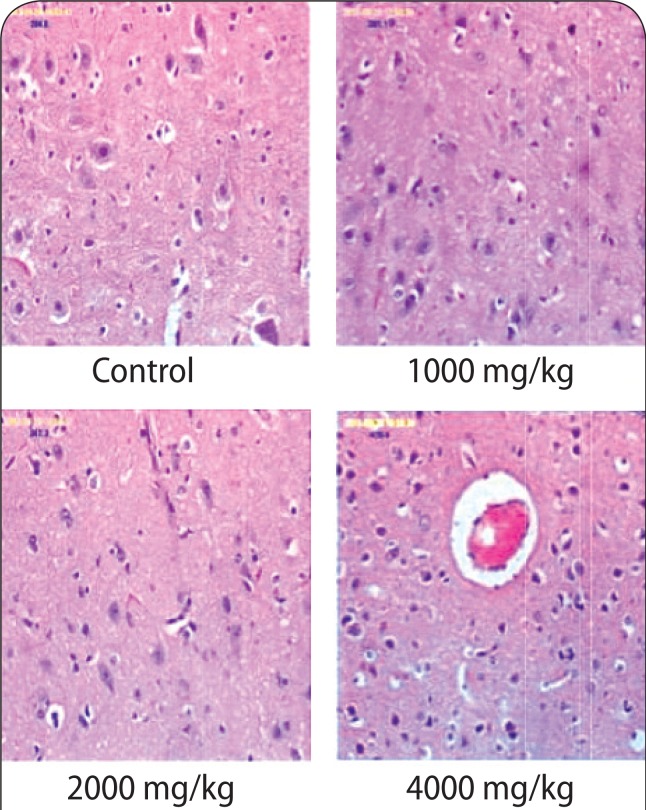
The micrographs of the brain sections obtained from untreated rats and rats treated with various doses of aqueous stem bark extract of *Bridelia ferruginea.* Magnification ×40.

**Figure 2:**
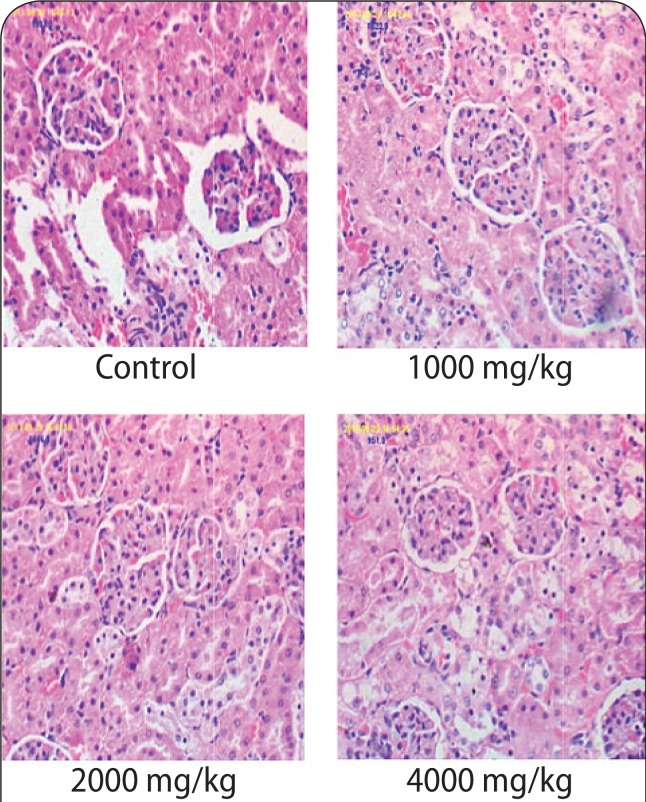
The micrographs of the kidney sections obtained from untreated rats and rats treated with various doses of aqueous stem bark extract of *Bridelia ferruginea.* Magnification ×40.

**Figure 3:**
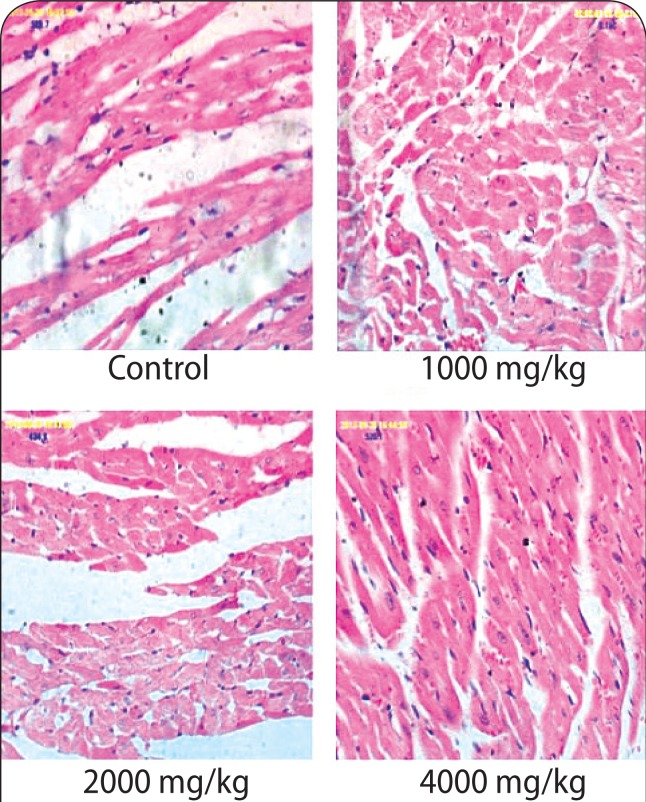
The micrographs of the heart sections obtained from untreated rats and rats treated with various doses of aqueous stem bark extract of *Bridelia ferruginea.* Magnification ×40.

**Figure 4:**
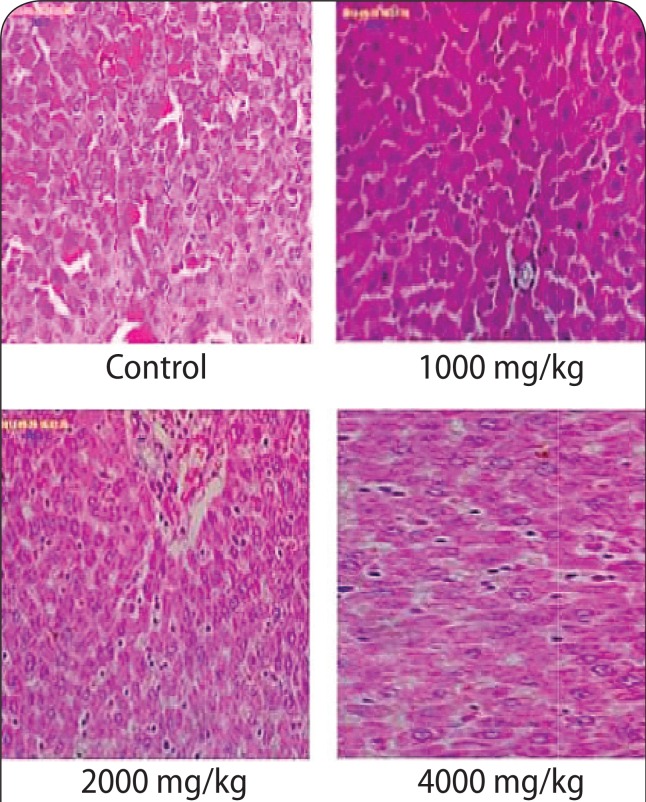
The micrographs of the liver sections obtained from untreated rats and rats treated with various doses of aqueous stem bark extract of *Bridelia ferruginea.* Magnification ×40.

The histology results of the brain in the 4 000 mg/kg group revealed cerebral edema (Figure 1). However, the histology results of the kidneys and heart at any of the doses applied (1 000–4 000 mg/kg) revealed no destruction to kidney and heart architecture (Figures 2 & 3). The liver histology results (1 000 mg/kg; 2 000 mg/kg) showed heavy lymphocytic infiltrates, congested sinuses and hemosiderin pigments (Figure 4).

## Discussion

Acute toxicity (LD_50_) testing has been widely used, though often criticized as a parameter for assessing toxicity (Lorke, 1983; Klaassen, 2001; Timbrel, 2002). This assay may provide information on the range of doses that could be used in subsequent toxicity testing; it reveals possible clinical signs elicited by the test substance or extract and is a useful parameter for estimating the Therapeutic Index (T.I) of drugs (Rang *et al*., 2001). The acute lethal effect of the aqueous stem bark extract of *Bridelia ferruginea* on rats produced no death within 24 hours of treatment either via oral or intraperitoneal routes. The oral minimum lethal dose (LD_50_) of the aqueous stem bark extract of *Bridelia ferruginea* was estimated as >4 000 mg/kg, implying the relative safety of the plant extract when administered acutely. Earlier studies by Bruce (1987) and the American Society for Testing and Materials (ASTME, 1987) established that any substance with LD_50_ estimate exceeding 4 000 mg/kg of body weight/oral route could be considered of low toxicity and safe in humans. The major signs of toxicity noticed within the first 2 hours included slight dullness in some of the animals at the higher doses administered (2 000 and 4 000 mg/kg) both by oral and *i.p* routes. The results further revealed that the extract caused an increase in the body weight of rats in the test groups, compared to the rats in the control group. Though the increase was non-significant, the weight increased throughout the 60-day period of administration, with a smaller increase at 2 000 and 4 000 mg/kg than at 1 000 mg/kg. The implication of this finding could be that the extract contained some nutrients (vitamins) that have the potential to increase body weight. Further studies may be done on this activity so as to explore all the weight increasing potentials of this extract and also determine the influence of doses on weight increase. Increased or decreased organ weight was observed as a sensitive indicator of organ toxicity by known toxicants (Dioka *et al*., 2002). The aqueous stem-bark extract of *Bridelia ferruginea* was found to cause a non-significant increase in the weight of harvested organs of rats (liver, heart and kidneys). Since this is a non-significant increase in organ weights, by extrapolation and implication, the results may be an indication of the low toxicity and relative safety of the extract.

Toxicity testing is relevant to risk evaluation as changes in the hematological system have higher predictive value for human toxicity when data are translated from animal studies (Olson *et al*., 2000). The various hematological parameters investigated in this study are useful indices that can be employed to assess the toxic potential of plant extracts in both man and animals (Sunmonu & Oloyede, 2010). According to Degruchy (1976), RBC and Hb are very important in transferring respiratory gases. Thus, in this study, the non-significant effect on RBC and HGB in the test groups, compared to the control, implies that there was no change in the oxygen-carrying capacity of the blood and amount of oxygen delivered to the tissues following the administration of various doses of the extract to the test animals. The increase in Hb is in contrast to the findings of Olarewaju *et al*. (2013) who reported a decrease in Hb in the test groups administered aqueous extract of *Bridelia ferruginea* stem bark, compared to control. PCV in this study showed a non-significant decrease at the highest dose (4 000 mg/kg) and an increase at the lower doses (1 000 and 2 000 mg/kg), compared to the control. This is in contrast to the findings of Olarewaju *et al*. (2013), who reported a decrease in PCV in the test groups at all doses of the administered aqueous extract of *Bridelia ferruginea* stem bark when compared to control.

MCV, MCH and MCHC, which are calculated, are of significance in diagnosing anemia in animals (Coles, 1986) and relate to individual red blood cells (Ashafa *et al*., 2011). The non-significant effect of the extract at the administered doses (1 000–4 000 mg/kg) on RBC and indices relating to it (HGB, PCV, MCV, MCH and MCHC) in the course of this experiment might be an indication that there was no destruction of matured RBCs. In addition, this observation may also be ascribed to the fact that the balance between the rate of production and destruction of the blood corpuscles (erythropoiesis) was not significantly altered by the extract. The result is also an indication that the extract lacks the potential to stimulate erythropoietin release in the kidney, which is the humoral regulator of RBC production (Polenakovic & Sikole, 1996; Sanchez-Elsner *et al*., 2004).

A reduction in platelet count in experimental animals has been reported to indicate an adverse effect on the oxygen-carrying capacity of the blood as well as on thrombopoietin (McLellan *et al*., 2003). This was however not the case in the present study as an increase in platelets was observed in the test groups compared to the control. This may be due to the stimulatory effect of the extract on thrombopoietin.

The decrease in WBC shown in Table 5 in the test groups compared to the control may imply that the aqueous stem bark extract of *Bridelia ferruginea* has the ability to cause immunosuppression. However, the significant (*p*<0.05) increase observed in WBC following the administration of the plant extract at 2 000 mg/kg may be due to increase in vascular permeability. Results also showed that other indices that relate to WBC (neutrophils and lymphocytes) were significantly increased at the highest dose administered (4 000 mg/kg) in the test groups compared to the control group; this may imply challenge on the immune system by the plant extract at much higher doses. According to McKnight *et al*., 1999, lymphocytes are the main effector cells of the immune system and the observed increase in the test groups compared to the control in this study may specifically be ascribed to the ability of the extract to stimulate neutrophils to promote phagocytosis (cellular ingestion of offending agents). High neutrophil counts can be the result of many factors that include bacterial infection, acute inflammation, stress response effect from some drugs and splenectomy, among others (Owoseni *et al*., 2010).

Cellular functions are altered when ROS generation exceeds the antioxidant defense, resulting in free radicals interacting with endogenous macromolecules (Muthukumaran *et al*., 2008). Malondialdehyde (MDA), a breakdown product, is a measure of lipid hydroperoxides, causing lipid peroxidation. MDA assay has been found to be a reliable predictor of oxidative damage and considered to be the most reliable marker of lipid peroxidation (Morrow, 2010). In the present study, there was a significant (*p*<0.001) increase in the level of MDA and a significant (*p*<0.001) decrease in SOD, GSH, GST and Vitamin C levels in the test groups (1 000–4 000 mg/kg) when compared to the control, which are signs of oxidative stress due to excessive formation of free radicals in the experimental animals. Srilaxmi *et al*. (2010) and Kalu *et al*. (2011) also made observations similar to the findings of this present study.

Alanine aminotransferase (ALT) and aspartate aminotransferase (AST) have been determined to be markers of hepatocellular injury while alkaline phosphatase (ALP) is a marker of cholestasis (Srilaxmi *et al*., 2010). Of the two, increase in ALT levels is a more specific indicator of liver injury because ALT catalyses the conversion of alanine to pyruvate and glutamate and is released in a similar manner. A non-significant decrease in AST, significant (*p*<0.001) decrease in ALT levels, as well as an insignificant (*p*>0.05) increase in ALP levels was observed in the test groups compared to the control (Table 7). It is possible to speculate that this observed effect of the extract was due to its ability to stabilize the plasma membrane and may be a potential in modulating the exogenous toxic effects of agents on liver cells. The findings in this study agree with the findings of a recent study conducted by Momoh *et al*. (2012) where the extract tested was observed to increase the level of ALT and decrease that of AST. The serum ALP levels are also related to the status and function of hepatic cells and according to the literature, an increase in serum ALP may occur as a result of increased synthesis, in the presence of increasing biliary pressure.

Urea, the end product of protein metabolism, and its concentration is influenced by the rate of excretion, while creatinine is the waste product of muscle metabolism. Renal diseases that diminish the glomerular filtration rate lead to urea and creatinine retention (Panda, 1999). The significant reduction (*p*<0.001) in the serum levels of urea and the non-significant reduction in creatinine in the extract-treated rats is an indication that the kidney is able to clear the waste products from the system; this is also indicative that the extract had no deleterious effect on a vital organ like the kidney. This finding is similar to that reported by Kolawole and Sunmonu (2010).

The total sperm count, motile sperm count and normal sperm morphologic features have been reported as indices of fertility in males (Small *et al*., 1987). The present study showed that the extract caused a non-significant decrease in motility and a significant (*p*<0.05) decrease in sperm count at 1 000 and 2 000 mg/kg, respectively, as well as an insignificant (*p*>0.05) increase in morphological aberration. Etta *et al*. (2012) and Verma *et al*. (2006) reported findings similar to the results in this study. However, this observation is paradoxical as higher doses did not decrease the sperm count.

Sharma and Agarwal (1996), in a research on the “role of reactive oxygen species in male fertility” revealed loss of motility and impairment of spermatogenesis, which is a pointer to the spermatotoxic effect of any extract (Sarathchandiran *et al*., 2007). The decrease in sperm count observed at the lower doses (1 000 and 2 000 mg/kg) could be attributed to the sperm entering the epididymis in a diluted form caused by lowered estrogen levels in the epididymis (Etta *et al*., 2012). This decreased sperm production may also be due to the extract directly suppressing the gonadal androgens resulting in sub-optimal testosterone levels at the lower doses. It is therefore possible to postulate that the effect of the extract on MDA elevation and reduction in SOD, GSH, GST resulting in reactive oxygen species production may have resulted in sperm quality damage in the present study.

Histological changes in the kidney, brain, heart and liver of the animals were also examined at the end of administration of the extract. The histology slides of the kidneys and heart at all doses (1 000–4 000 mg/kg) showed no destruction to kidney and heart architecture. The observation on the kidney, however, is contrary to the findings of Kolawole *et al*. (2009) who noted acute pyelonephritis with edematous infiltration of cells in rats fed with *Bridelia ferruginea* extract. This observation may be due to the effect of methanol (the extraction solvent used in the study), which has been shown to cause liver and kidney damage (Ezekwe *et al*., 2013). Ofogba *et al*. (1998) reported adverse effects upon examination of histopathological slides of the heart following administration of *Bridelia ferruginea* extract; a finding that is contrary to the effect observed on the heart in the present study following administration of the extract.

The liver histology results (1 000 mg/kg; 2 000 mg/kg; 4 000 mg/kg) showed heavy lymphocytic infiltrates, congested sinuses and hemosiderin pigments, which is in contrast to the study conducted by Bakoma *et al*. (2013) who reported no obvious histological changes observed in organs of *B. ferruginea* extract treated animals compared to controls. This observation may be due to the variation in the length of administration of the plant extract. However, the biochemical liver enzyme biomarkers of animals treated with *B. ferruginea* were normal.

### Conclusion

The relative safety of the aqueous stem bark extract of *Bridelia ferruginea* as obtained in the present study is comparable to the report of Bakoma *et al*. (2013). However, the present study revealed the potential of this extract to cause lipid peroxidation and subsequent damage of sperm quality. Hence the extract of *Bridelia ferruginea* must be rarely used and if its use is inevitable, antioxidant regimen must be incorporated.
